# The Relation of the University to the Medical School*Address delivered before the Joint conference of the Council on Medical Education and the Committee on Medical Legislation of the American Medical Association, Chicago, Feb. 28 to March 2, 1910.

**Published:** 1910-06-15

**Authors:** Jacob Gould Schurman

**Affiliations:** President of Cornell University, Ithaca, N. Y.


### THE RELATION OF THE UNIVERSITY TO THE MEDICAL SCHOOL.*

JACOB GOULD SCHURMAN.
President of Cornell University, Ithaca, N. Y.

The American people are universally distinguished for their business ability and business successes. We live, however, in a world of universal compensations and retributions. If we have an enormous business talent which has won for us a name in the field of trade and commerce, we tend to draw into this province all our other interests. The pulpit and the press are not infrequently denounced as organs of syndicated wealth. In this very city of Chicago, the colleges and universities of the country have recently been declared worthless by a multimillionare citizen because their graduates have not been trained in the ways of money making. And if in this presence I do not say that the medical profession has been commercialized I do not hesitate to assert that many medical schools and colleges have been established for the pecuniary benefit of their promotors, with the result that we now have in the United States almost as many of these institutions as all the rest of the civilized world, and our standards of medical education are a disgrace to the nation and an outrage on humanity.

The personal profit of individuals has been the most powerful and prolific motive for the multiplication of medical colleges, as it has also been the most pernicious. Fortunately, however, the general spirit of the age, as well as higher ideals of professional education, are working against these commercial medical schools. Not only are the motives of their founders and supporters better understood,

*Address delivered before the Joint conference of the Council on Medical Education and the Committee on Medical Legislation of the American Medical Association, Chicago, Feb. 28 to March 2, 1910.

but the superiority of medical schools founded on a different basis throws them more and more in the shade, while the growing costliness of a sound education in modern medicine makes their puny efforts ridiculous. At the same time there has been a growing appreciation of the fact that every professional school to be vital and efficient must be a department of a university. Under the influence of these ideas and agencies I have no doubt that the number of private-enterprise medical colleges is destined rapidly to disappear in this country. And I need scarcely say to this audience that this process is already hopefully under way.

I turn to a second motive for the establishment of medical colleges which deserves more consideration than it has hitherto received. I am, indeed, firmly persuaded that no mēdical school of the highest class can be maintained apart from a university. But I am equally persuaded that no university, merely because it is a university, is justified in founding a medical college. There has been a general impression not only in university circles, but among the members of the medical profession, that a university needed a medical department to round out its organization, and, as it were, to complete its existence. This belief is without warrant in history. It is true that the oldest university in the world, the University of Salerno, had a medical school, and was, indeed, nothing but a medical school. But within a generation came the universities of Bologna and of Paris, the one famous for law and the other for theology, and neither of them in the days of their first glory possessed a medical department. In his great history of universities Rashdall shows that some of the most famous of these institutions had only one or two departments, and that no particular faculty, medicine or any other, can be regarded as essential to the idea of the university, as that institution developed among the nations of Europe. The word university has nothing to do with universal knowledge. It means, on the contrary, merely a guild or corporation. When the founder of Cornell University declared that he would found an institution where any person might find instruction in any study, he formulated a brand-new idea of a university. Yet even at Cornell that idea remains at best an ideal, so vast is the sum total of human knowledge, so varied the fields of investigation, and so costly the miantenance both of instruction and research. And without special endowments, and large endowments too, neither Cornell nor any other university would be justified in the establishment of a medical department. The appeal that such a department is necessary to make a complete university is, as I have shown, historically without justification. And in any event to establish a university department in medicine which you have no special funds to maintain is to be a party to fraud on the public. This is especially true at the present time when, owing to the advance of science and the indispensableness of making provision for it in the curriculum, medical education is the costliest education given at our universities. I recall with satisfaction that on different occasions before the establishment of our own medical college at Cornell University, when I was solicitedto"a dopt"this or that medical college as the medical department of Cornell, I always replied that I did not want a medical department unless it were as organic a part of the university as any other college; nor would I favor the establishment of one without adequate special endowments for its maintenance. I may add that the members of the medical profession with whom I dealt were invariably amazed at the position I took, and in some cases felt it an affront to themselves that I was so mercenary and autocratic as to decline the offer they made me of eminent men unless the university had the right to control them and endowments to support their work! These recollections are from twelve to eighteen years old. And when I say that the position which I took in those years is now the position of the leaders of the medical profession, I may be pardoned for finding in that circumstance a striking evidence of the great advance which has been made in so short a time on the subject of medical education.

I have spoken of the costliness of medical education. In no other field has science brought such a wealth of new and fruitful discovery. All scientific work is expensive, and the medical sciences the most expensive of all because they deal with the complex phenomena of human life. All education in engineering science is also expensive. But engineering rests in a last analysis on the sciences which deal with energy and motion. And these sciences are simplicity itself in comparison with those which deal with the mysteries of living organisms. Education in agriculture, which has to do with soil, and plant, and animal, is more expensive than engineering education. But, as I have already said, medical science, dealing with life in its highest form, is the most expensive of all. It is to be added that its costliness is likely to increase. For both the discovery of new scientific facts and principles and the application of these to medical practice will call for research on the part of able men who must devote to it the uninterrupted service of a lifetime and the maintenance of large and well-equipped laboratories for the conduct of their experiments and investigations. And even scientific investigators, teachers, and laboratories, do not complete the list of objects of expenditure in a medical college. It is now coming to be accepted as a commonplace that a medical college is in as much need of a hospital for the study of disease as it is of laboratories for scientific investigations and experiments. And at the first annual conference of this Council on Medical Education Dr. Councilman of Harvard stated that the hospital must be regarded in a certain sense as a laboratory for the study of disease, and that the future and the highest development of medical education in this country is wrapped up with the control of hospitals. I unders'ood that a thousand dollars a bed is a moderate estimate of the cost of maintaining a hospital after the institution has been built and equipped. A hopital for a medical college with

200 beds would, therefore, require for its maintenance, after it had been built and equipped, an endowment of $4,000,000 or $5,000,000. When a couple of years ago I recommended to the faculty of the Cornell University Medical School that we should restict admission to graduates in arts or science of recognized colleges and universities, I stated that the faculty should not accept this recommendation unless they were prepared to see the enrollment in the medical college drop from 300 or 400 students to 50 or 100 students. But I added that we should not decrease, but rather increase the cost of maintenance. And the cost per student is going to be between $2,000 and $3,000. Of course, this expenditure could be reduced by diminishing the number of men on the faculty who give their entire time to investigation. On the other hand, it would be increased if our medical college were put to the expemse of supporting a hospital of its own.

In view of these facts, the first advice I should be disposed to give to a university that was inclined to "round out its organization" by establishing a medical department would be the single word: *don't.* Institutional ambition is as unhallowed a motive for the multiplication of medical colleges as the love of personal gain. The latter motive has filled the country with commercial schools of medicine. These schools are a disgrace to our country. Starving medical departments of impoverished universities are equally disgraceful. And the supposed glory accruing to a university which institutes professional schools when it has no funds to maintain them is as dishonorable a motive as the gain in professional advancement sought by physicians and surgeons who establish and maintain the private-enterprise medical institution.

The first service, therefore, which universities may render to medical education is a negative one. But, though negative, it is fundamental and vital. Have done with sham in medical education! Recognize that a medical department is the costliest department of a university! There may be a first-class university without a medical department. But a medical department which is not first class lowers the standing and hurts the reputation even o fa good university. Whether any given university shall found a medical school or not is not a difficult question to determine. If the university has ample funds for this purpose which it ought not or cannot use for other departments to which it is already committed, if there is no first-class medical college in its state or locality, if, in a word, the university has both the opportunity and the funds let it by all means establish a medical department. But let this new institution be not an attachment or (detachment) to the university, but an organic and vital member like other departments of the university, and ,like them, animated by its spirit and controlled by its standards. A genuine university department of medicine of this character is as likely as any other department to receive endowments from the public for the maintenance and extension of its work. Indeed, I am inclined to think at the present time that no other object embraced in a university curriculum appeals so strongly to the interests and sympathies of public-spirited and generous men and women of wealth as the problem of the prevention and cure of disease, which is the central function of the medical faculty of the university.

Besides putting the medical school on a proper and dignified basis, the university can render to this department another service of exceeding great importance. Men engaged in the active practice of any profession are apt to lose touch with educational ideals. Like the world about them, they want practical results and quick methods. The scholars and scientists, however, know that in educational matters the shortest distance even to a practical goal is not always a straight line. In order to be a capable physician or surgeon, a man needs to know a good deal more than the "practical" branches of the curriculum. Sciences apparently remote from those branches often illuminate them with new light. The facts which stimulate and fructify the mind of the investigator are often out-of-the-way, trivial and insignificant facts. Nothing in the life of Darwin has impressed me more than this very circumstance. Why, Darwin owed to the reading of a work on economics the germinal idea of his whole theory of evolution. Technical and professional education is narrow and arid unless interfused with a broad and generous culture in the sciences, and also in the liberal arts, and this is peculiarly true of medical education. So far as the sciences are concerned, the leaders of the medical profession are, I am glad to say, keenly alive to their needs. Besides good general training in physics, chemistry and biology—training which in most cases must be furnished by colleges and universities, as only a few high schools are equipped to supply it—these leaders are demanding thorough training in anatomy, histology, embryology, physiology, physiologic chemistry, pharmacology, pathology, and bacteriology. If to these indispensable sciences be added a reading knowledge of French and German, which is an almost indispensable tool for the medical man, it will be seen that a medical education demands a good training in the sciences and presupposes also a certain amount of linguistic discipline.

I have been specifying the scientific requirements of medical curriculum from the point of view of medicine alone. But a university cannot be content to turn out men with merely a professional training. Physicians and surgeons are not mere practitioners; they are also human beings. And a university must think of training them for manhood and citizenship, as well as for the practice of medicine or surgery. Now we have reached a point in the higher education of this country in which it is recognized that the proper time to begin professional work or to specialize with reference to that professional work is at the end of the sophomore year in college. I have no time here to argue this position. But I may say that it is a conclusion which has been reached both by the National Association of State Universities and by the Association of American Universities; the former organization embracing all the state universities of the country, and the latter a score or so of our foremost universities, both state and endowed. Perhaps the position of the Association of American Universities would be more accurately described if I stated that its desideratum is a minimum of two years of college work as a basis for professional work, and that some of the institutions embraced in that association would prescribe even a longer time The important point is that both these organizations, which include all the leading universities of the country, demand that at least two years shall be spent on liberal education before undertaking professional education or specializing with a view to it. I am myself in hearty accord with this program. I should like to see our schools of medicine, law, and engineering reorganized with reference to it. And I am glad to say that a movement of that kind is now in full force in our schools of law, and especially in our schools of medicine. While Johns Hopkins in 1893, Harvard in 1900, and Cornell in 1908, demanded a degree in arts or science of students in medicine, a score or more of medical schools now require two years of college work and about the same number now require, or are soon to require, one year of college work which shall include the courses in physics, chemistry and biology.

So far as this latter group is concerned, the requirement of a year of college work is probably due to the necessity of providing satisfactory instruction in physics, chemistry and biology. But as this departure breaks with the traditional high-school requirement for admission to the medical course, it prevents the high schools from continuing to be feeders of the medical colleges, and yet it does not establish a preliminary education sufficiently high to command the con: fidence and the patronage of college men. The chances are, therefore, that this arrangement will prove only a provisional one, and the institutions which have adopted it will either rescind their action and re-establish their connections with the high schools or prescribe a preliminary education of two years in arts and sciences, with a view to attracting the patronage of college undergraduates. It has not, I think, been sufficiently considered that when a medical college once severs its educational connections with the high schools it may so far as the effects on attendance are concerned almost as well prescribe two years of college work as one, and pretty nearly as safely three years as two.

Another service which the universities should render to the medical college is the determination of the question, What subjects of study should enter into the education of prospective physicians and surgeons? Assuming, that is to say, that candidates for the medical degree will have at least two years of study in a college of arts and sciences, the question is, What subjects shall they study? The criterion in answering this question should undoubtedly be the requirements of a liberal education. And it frequently happens that in the case of a professional subject like medicine the requirements of a liberal education in arts and sciences coincide to some extent with the scientific conditions which the medical profession would like to see fulfilled before students enter the medical college. I believe that a perfectly satisfactory adjustment of the problem can be worked out along these lines. I am well aware that this has not been the prevalent point of view. On the contrary, we have had two extreme positions more or less contradictory, which seemed to exclude a middle ground on which educators and medical men might alike agree. These two conflicting views have been voices by the advocates of liberal education on the one side and of professional education on the other. The former have been in the habit of insisting that an A. B. degree should be a condition of admission to a medical college without much regard to the subjects embraced in the A. B. curriculum, provided only that a liberal education might be claimed on the basis of that A. B. degree. I believe that the underlying desideratum of these advocates was a classical education.

Opposed to them stood the other party made up mostly of medical men who insisted that an A. B. course in itself was of very little value as a preparation for medical studies. What they desired was a preliminary education in the pure sciences: physics, chemistry, biology, comparative anatomy, etc., on which the medical sciences were based. Between these two schools there has in the past been little agreement. But I believe that the time has come for a satisfactory adjustment of the problem.

I have already stated that the foremost educators today agree in demanding that the first two years of the college course shall be required of students who intend to enter such professional schools as law and medicine. These educators, furthermore, have in the course of the last two years come to a pretty close agreement as to the content of the curriculum of these two years of college study. They are agreed that it should embrace English, history, economics, one or two foreign languages, and mathematics, and one or more of the following sciences: physics, chemistry, biology, geology or botany. Such a curriculum furnishes the prospective medical student not only with subjects which are requisite to a liberal education, but also with subjects which form the best preparation for the subsequent study of medicine. Do you ask me what studies I would prescribe for this prospective medical student? I answer at once the English language and literature, French and German, history, economics, advanced algebra and trigonometry, and chemistry, physics, and biology. To these I would add a subject which I never see mentioned by advocates of either of the extreme positions I have already described. I mean the important subject of the study of the mind, and more particularly those aspects of it which are embraced in the modern science of experimental psychology. Considering the close relation between mind and body and the dependence of some diseases on mental conditions, I am often amazed that medical men fail so completely to realize the importance of the study of the sciences of mind as a part of that curriculum of the preliminary education they lay down for prospective students of medicine. I believe that in the first two years of a college course there is time to include psychology with the other studies in liberal arts and sciences which I have already specified. And I do not hesitate to assert that a young man who has received a thorough training in these subjects may, on the one hand, be regarded as a liberally educated man, and, on the other, as scientifically qualified for admission to a medical college and the successful study of the sciences prescribed in the first year of the medical curriculum.

The last service I shall mention which a university could and should perform for a medical college is to provide it with capable and devoted teachers and investigators imbued with the spirit of researsh. I do not think that the medical profession, and I am sure that the general public does not realize the nature of the change which the advances in modern science make necessary in the faculties of our medical schools. In the commercial and private-enterprise medical colleges, a number of practicing physicians and surgeons came together and devoted the fag end of their time to the instruction of students in medicine. Not only were these men not scientifically prepared for the task they assumed, but their interests lay in the practice of their profession, and the one object they had in view in establishing a medical school was to enlarge their medical practice and make it more lucrative. Now the authorities of our universities know that we cannot have efficient teachers and fruitful investigators, first, unless they are thoroughly trained for their work, and, secondly, unless they devote their whole time and energy to that work. The staffs of our medical colleges are sadly in need of reorganization under the guidance of this criterion. If our medical colleges are to rank with other colleges in the university, it can only be because the teachers and investigators on the staff bring undivided hearts to their work and render the same whole-souled service. It is practically impossible for a man to have a large and successful practice and to be at the same time a scientific and efficient teacher and investigator in medicine. I lay it down, therefore, as a first condition for the elevation of our medical colleges, that their chairs shall be filled by teachers and investigators of the highest scientific standing, who devote their entire time and energy to the work of instruction and investigation. There is no difficulty in applying this criterion to the professorships represented by the first two years of the medical curriculum—to subjects like anatomy, physiology, biochemistry, pathology, bacteriology, etc. I recognize, however, that there is a difficulty when we come to the professional and practical branches embraced in the last two years of the curriculum—medicine, surgery and obstetrics, not to speak of the minor specialties. Yet I am confident that it is impossible, under the present system of appointing to these subjects distinguished practitioners who continue in unrestricted practice, to produce satisfactory results in the work of instruction and investigation in these fields. I recognize, on the other hand, that men are not qualified to teach medicine, surgery, obstetrics, children's diseases, and other special diseases, unless they are engaged in the treatment of these diseases. It looks, therefore, as though a satisfactory solution of the problem of thorough and scientific instruction and investigation, both in the major and minor practical branches of the curriculum, would be an impossibility. I hope at any rate that I have stated the problem and shown the supreme importance of finding a solution. No solution will be satisfactory which does not do justice to the inexorable demands made by modern science on the teacher and investigator, and at the same time to the necessity of constant pratical experience as a qualification for professorships in the pratical branches of the curriculum. If I may venture to make one suggestion in the way of a positive solution of the problem I have stated, it would be that future professors in medicine, surgery, obstetrics, and similar branches in our best medical colleges will give a fixed portion of their time and energy to the work of instruction and investigation in the medical college. I should not be content with their evenings or even with their afternoons. Teaching and investigation demand not only their best powers but their best powers when they are fresh and capable of their highest exercise. Keeping this consideration in mind, I would suggest that their forenoons might be devoted to the work of the medical college. And if the rest of the day were devoted not to general practice, but to a consulting practice, or to practice in a hospital owned or controlled by the medical college, we should have the best solution of the problem I can think of at the present time.

I have been speaking of the services which a university can and should render a medical college. I have spoken also of the unsatisfactory condition in which much of our medical education is at the present time. But I cannot conclude without calling attention to the enormous progress which has been made in the last few years, and I want finally to express my own belief and conviction that there is no subject embraced in the purview of our universities in which such rapid progress in recent years has been made, both in instruction and investigation, as in medicine, and there is no subject in which I look toward the future with more hopefulness and confidence of results, great, fruitful, and beneficial to mankind.—*A. M. A. Journal*, April 16, 1910.

